# Development and validation of COVID-19 Impact Scale

**DOI:** 10.1186/s40359-022-00793-w

**Published:** 2022-04-04

**Authors:** Haewon Min, Jinwon Kim, Kibum Moon, Seungjin Lee, Jin-young Kim, Young-gun Ko

**Affiliations:** 1 School of Psychology, Korea University, 145, Anam-ro, Seongbuk-gu, Seoul, 02841 Republic of Korea; 2 Office of Digital Information, Korea University, Seoul, Republic of Korea; 3 Department of Child Studies, Seoul Women’s University, Seoul, Republic of Korea

**Keywords:** Impact of COVID-19, COVID-19 pandemic, Mental health, Scale development, Scale validation, Factor analysis

## Abstract

**Background:**

As the COVID-19 (Coronavirus disease 2019) pandemic is prolonged, psychological responses to the pandemic have changed, and a new scale to reflect these changes needs to be developed. In this study, we attempt to develop and validate the COVID-19 Impact Scale (CIS) to measure the psychological stress responses of the COVID-19 pandemic, including emotional responses and difficulty with activities of daily living.

**Methods:**

We recruited 2152 participants. Participants completed the CIS, the Fear of COVID-19 Scale (FCV-19S), and other mental health related measures. The factor structure, reliability, and validity of the CIS were analyzed. In addition, the validity of the scale was confirmed by its relationships to the existing measures assessing fear of COVID-19, depression, anxiety, subjective well-being, and suicidal ideation.

**Results:**

Using exploratory factor analysis (*N*_*1*_ = 1076), we derived a one-factor structure. In confirmatory factor analysis (*N*_*2*_ = 1076), the one-factor model showed good to excellent fitness. The CIS was positively correlated with depression, anxiety, suicidal ideation, fear of COVID-19 and negatively correlated with subjective well-being. The FCV-19S did not show significant correlations with subjective well-being or suicidal ideation, and FCV-19S’s explanatory powers on depression and anxiety were lower than those of the CIS.

**Conclusions:**

These results support that the CIS is a valid assessment of emotional problems and deterioration of the quality of life caused by the COVID-19 pandemic. Finally, the limitations of this study and future research directions are discussed.

## Background

The Coronavirus disease 2019 (COVID-19), first reported in December 2019, has spread worldwide at unprecedented speed and scale. The World Health Organization (WHO) declared it a Public Health Emergency of International Concern in January 2020 and a pandemic in March [58]. By December 2021, 2 years after its first identification, COVID-19 is still ongoing around the world , resulting in more than two hundred million confirmed cases and five million (as of December 23, 2021, [25]).

The protracted pandemic with no sign of near-term ending has affected all areas of society, including economy, industry, education, and culture. To respond to these challenges posed by the pandemic, various issues have been addressed, such as prevention and vaccine development (e.g., [1, 5, 22, 23, 31]), vaccine donation and non-adopters (e.g., [52, 53]), socio-economic implications (e.g., [40, 49]), online teaching (e.g., [27]), and campus preventive measures (e.g., [57]).

It is predictable that the pandemic poses a continuing challenge to public health, including physical health and mental health. Recent studies have reported that people experience various psychological difficulties during the COVID-19 pandemic, ranging from severe symptoms to stressful responses such as depression, anxiety, suicidal ideation, concern about infection, uncertainty and helplessness from the prolonged pandemic, and loneliness from quarantine and social isolation [2, 42, 54]. Some researchers have suggested that if these psychological difficulties persist without being identified or treated, they can lead to more serious psychological problems and chronic psychopathology [8, 13, 60]. Therefore, it is necessary to understand what adverse psychological effects and to what extent they are experiencing during the pandemic to prevent such psychological problems.

Although some previous studies have identified adverse psychological effects under pandemic conditions, most of these studies were based on a relatively short period of pandemic (severe acute respiratory syndrome in 2003, first wave of COVID-19) or limited subjects (healthcare workers, confirmed patients) (e.g., [14, 19, 21, 34, 35]). A thorough comprehension about what psychological difficulties the general population experience and how these are changing as the pandemic continues is lacking [43]. The current pandemic has become exceptionally prolonged and universally experienced, making the daily life of the general population upended and day-to-day exhausted [4]. Thus, it is necessary to routinely confirm and track psychological problems in the midst of the open-ended pandemic. To facilitate this, a reliable and valid assessment instrument that enables easy repeated administration to the general population while reflecting features of the current pandemic is essential.

There are some specific scales to assess psychological problems from the COVID-19 pandemic. Scales developed in the early days of the pandemic focus more on pathological responses, such as fear [3], phobia [7], anxiety [41, 55], and trauma [44]. These all reflect acute stress responses known to be common in the early pandemic phase. However, it has been reported that responses are changed as the pandemic is prolonged. A mental health survey conducted across five waves from March 2020 in the United Kingdom found that while people said they still suffered from the pandemic, levels of anxiety and worry they reported gradually declined [36]. A study using the Fear of COVID-19 Scale [3] also reported that the level of fear peaked at the first wave in April 2020. However, only 5.3% reported an increase since the beginning of the pandemic [28]. The World Health Organization European Office found that anxiety and stress were mainly reported immediately after the pandemic outbreak. However, as time goes by, life functioning problems (such as daily routines, interpersonal relationships and occupational activities) and various negative emotions (such as loneliness, anger and irritation) are increasingly reported [59]. This suggest that, as the pandemic is prolonged, psychological problems experienced by the public are expanding to a wide range including various negative emotional responses as well as difficulties in daily functioning.

Accordingly, some scales have been recently developed to evaluate psychological problems based on stress responses in that the prolonged pandemic could also be understood as a type of stressor which can be appraised as demands taxing or exceeding one’s adaptive capacity [60]. Operationally, studies of psychological stress have focused on either the occurrence of environmental events that are judged as taxing one’s ability or individual responses to stressful events [18]. Stress response refers to a set of affective, cognitive, somatic, and behavioral manifestations within the range of functional integrity, in contrast to dysfunctional and morbid psychopathology which is not sensitive below the critical diagnostic threshold [29]. Therefore, when measuring and studying stress responses, the composition may vary depending on whether the stressor is focused on and/or which factors among emotional, cognitive, physical, and behavioral factors of stress responses are focused on.

Extant scales are based on evaluating the degree of stressfulness for each COVID-19 relevant stressor [43] or measuring only emotional stress responses to COVID-19 [8, 60]. In the development of the scale, we intend to reflect that there are increasing reports of difficulties in daily living from previous mental health surveys and that appraisal aspects of own functioning are also important along with emotional responses. Thus, we tried to compose perceived difficulty in daily life functioning as well as various negative emotional responses, trying to organize them into a compact scale as much as possible.

## Method

### Procedure for development of the COVID-19 Impact Scale

The aim of the current study was to develop and validate a new scale to examine the psychological effects of the COVID-19 pandemic. The COVID-19 Impact Scale (CIS) was developed through two separate stages. In the initial stage, we reviewed the literature on the psychological effects of stressful events and the COVID-19 pandemic. Since most of the papers on the psychological effects of the COVID-19 were based on the data at the beginning of the pandemic, we also reviewed related reports and announcements published by trusted sources such as government institutions or major news media to investigate its prolonged effect. Based on these reviews, we collected keywords related to negative emotions and life functioning problems that can be experienced with prolonged pandemic (e.g., irritation, fatigue, marital problems, feeling of isolation).

In the next stage, we evaluated a total of 31 pooled keywords through discussions among the authors (comprising master’s graduates, doctoral students, and professors in clinical psychology). After removing those keywords with similar content or relatively low relevance, we left 12 keywords. With these keywords, we generated items asking the subjective impact of the COVID-19 pandemic. Each item measures the degree of each distress caused by the COVID-19 pandemic, which is based on a 5-point Likert scale: None (0), mild/rarely (1), moderately/sometimes (2), severe/often (3), very severe/very often (4). The generated 12 items were sent to an expert panel (comprising two licensed clinical psychologists and two professors in clinical psychology) to get the review. After two items were deleted based on the feedback received from the expert panel, the final 10 items were included in the CIS (see “Appendix” for the items).

### Participants

A total of 2152 participants took part in a university’s mental-health survey conducted in July 2020. Participants were recruited via an email advertisement. Before conducting the online survey, participants were given information that their responses could be used for research. All participants voluntarily signed a written informed consent form. They completed a set of self-reported questionnaires via an online survey platform. The average time to fill out the questionnaires, with a total of 77 questions, is 10–15 min. All procedures were approved by the Institutional Review Board of the university at which the study was conducted.

### Measures

#### The COVID-19 Impact Scale (CIS)

We developed the CIS, which was composed of 10 items measuring the psychological effects of the COVID-19 pandemic (e.g., “How often are you experiencing irritation regarding the COVID-19 related problems currently?” and “How much do the COVID-19 related problems interfere with your interpersonal relationships?”). All items are rated on a 5-point Likert scale, ranging from 0 (none) to 4 (very severe / very often). The internal consistency of the CIS was 0.91.

#### The Fear of COVID-19 Scale (FCV-19S)

The FCV-19S [3] is a 7-item measure to assess how threatened or worried people are about the COVID-19 pandemic. All items are rated on a 5-point Likert scale, ranging from 1 (strongly disagree) to 5 (strongly agree). Because this scale had no Korean version at the time this study was conducted, independent Korean translation and back-translation were done by bilingual graduates. The final questionnaire was then confirmed through discussions with researchers. The mean of the Korean version of FCV-19S was 16.09 (*SD* = 4.72) with a range of 7 to 35. It was lower than the pooled mean of FCV-19S (*M* = 18.57) from the meta-analysis [32]. It was also lower than the mean of the Asian continent (*M* = 18.36) and college students (*M* = 17.95) reported in the same paper. The reliability of the Korean version was good, Cronbach's Alpha = 0.81.

To explore the factor structure of the FCV-19S, factor analysis with maximum likelihood method was conducted. Two factors were extracted with eigenvalues of more than one, which accounted for the cumulative explained variance of 49.49%. It was different from the result of the original validation paper, which reported a single factor model [3], but consistent with the results of some subsequent validation studies, which reported a two-factor structure model (e.g., [11, 16, 24, 33, 37, 47]). The items classified for each factor also appeared the same as the previous studies, which reported the presence of two factors: the first factor related to symptomatic expressions of fear (items 3, 6, and 7, factor loadings = 0.58–0.82) and the second factor related to emotional fear reactions (items 1, 2, 4, and 5; factor loadings = 0.52–0.72).

#### The Center for Epidemiologic Studies Depression (CES-D)

The CES-D [46], a widely used screening instrument for depression, was designed to measure depressive symptoms in the general population. The scale comprises 20 items assessing frequency of depression symptoms experienced in the previous week on a 4-point Likert scale, ranging from 0 (rarely or none of the time) to 3 (most or all of the time). A Korean version of the CES-D was used, and its internal consistency, Cronbach’s *α* = 0.91, and validity were reported to be as good as those of the original scale [17]. Internal consistency in the present study was good, *α* = 0.94.

#### Generalized Anxiety Disorder 7-item (GAD-7)

The GAD-7 [51] was developed to assess the severity of generalized anxiety. It was reported to be a valid and efficient tool for screening for generalized anxiety disorder. Participants were asked how often they had been bothered by seven symptoms of generalized anxiety disorder during the previous 2 weeks. All items are rated on a 4-point Likert scale, ranging from 0 (not at all) to 3 (nearly every day). The internal consistency of the original GAD-7 was excellent, Cronbach’s *α* = 0.92 [51], and the internal consistency of this study was also excellent, *α* = 0.90.

#### Mental Health Continuum-Short Form (MHC-SF)

The MHC-SF [26] is a 14-item short form of the Mental Health Continuum-Long form. It assesses the degree of subjective well-being on a 6-point Likert scale, ranging from 0 (not at all) to 5 (every day). A Korean version of the MHC-SF was used, and its internal consistency was reported to be excellent, Cronbach’s *α* = 0.93 [30]. Internal consistency in the present study was also excellent, Cronbach’s *α* = 0.93.

#### Scale for Suicide Ideation (SSI)

To measure the current intensity of suicide ideation, we used the SSI [50]. This scale was originally designed to be administered by clinicians [10] and was modified to be a self-report questionnaire by Shin et al. [50]. The scale comprises 19 items, all of which are rated on a 3-point Likert scale, ranging from 0 to 2. The reliability of the modified questionnaire was good, Cronbach’s *α* = 0.87 [50]. Internal consistency in the present study was also good, Cronbach’s *α* = 0.85.

### Data analysis

We used descriptive statistics to describe the basic features of the participants. We randomly split the dataset in half to conduct both exploratory factor analysis (EFA) and confirmatory factor analysis (CFA). In the EFA (*N*_*1*_ = 1076), we explored the dimensionality of the scale and item loading, adopting a principal axis factoring analysis with oblique rotation. In the CFA (*N*_*2*_ = 1076), we validated the proposed model in EFA using parceled items. We did correlation analysis to confirm the validity through the relationships with existing mental-health measures. All data analyses were conducted using the R program [45].

## Results

### Descriptive statistics

The sample consisted of 2152 participants (males = 47.82%, females = 51.81%, other = 0.37%). Age ranged from 17 to 52 years (*M* = 23.94, *SD* = 4.53), and 61.20% were undergraduate students (year: 1^st^ = 16.64%, 2^nd^ = 11.85%, 3^rd^ = 15.20%, 4^th^ = 15.01%, graduated = 2.51%) and 38.80% were graduate students (master’s students = 21.84%, Ph.D. students = 16.96%). Table 1 shows the descriptive statistics and internal consistency of the CIS across the whole sample (EFA sample and CFA sample).

**Table 1:** Descriptive statistics of the COVID-19 Impact Scale (N = 2152)

Item	EFA Sample^a^ (*N*_*1*_ = 1076)			CFA Sample^b^ (*N*_*2*_ = 1076)		
	*Mean (SD)*	*r*_*tot*_	*α if item deleted*	*Mean (SD)*	*r*_*tot*_	*α if item deleted*
Item 1	2.17 (0.94)	.69	.89	2.12 (0.93)	.69	.90
Item 2	2.09 (0.99)	.78	.89	2.07 (1.02)	.77	.89
Item 3	1.90 (0.86)	.56	.90	1.85 (0.88)	.60	.90
Item 4	1.93 (0.87)	.81	.89	1.89 (0.90)	.80	.89
Item 5	1.71 (1.02)	.75	.89	1.71 (1.04)	.75	.90
Item 6	1.16 (1.07)	.68	.89	1.10 (1.10)	.74	.90
Item 7	1.97 (0.95)	.76	.89	1.98 (0.98)	.76	.90
Item 8	1.49 (1.01)	.74	.89	1.50 (1.04)	.74	.90
Item 9	1.97 (1.12)	.60	.90	1.85 (1.10)	.60	.90
Item 10	2.14 (1.16)	.61	.90	2.07 (1.18)	.65	.90
Cronbach’s alpha	.90			.91		

*r*_*tot*_ = corrected item-total correlation

^a^Skewness coefficients =  − 0.15 to 0.65; Kurtosis coefficients =  − 0.90 to 0.28

^b^Skewness coefficients =  − 0.03 to 0.82; Kurtosis coefficients =  − 0.88 to 0.16

### Exploratory factor analysis

The whole sample was split into two randomly selected halves to examine the factor structure of the CIS. We used the first half of the sample (*N*_*1*_ = 1076) for an exploratory factor analysis. The significance of Bartlett’s test of sphericity (*χ*^*2*^(45) = 5382.83, *p* < 0.001) and the Kaiser–Meyer–Olkin measure of sampling adequacy (KMO = 0.93) indicated adequacy of the data for applying the EFA. We conducted the EFA using the Principal Axis Factoring analysis with oblimin (oblique) rotation, which allows factors to be correlated. The number of factors was determined by a combination of the Empirical Kaiser Criterion [12], the scree plot [15], and the minimum average partial test [56]. The analysis revealed a single factor under a cutoff of the Eigenvalue 1, explaining 49.39% of the total variance. We found that communalities for all items exceeded 0.3, and all items significantly loaded on the factor using exclusion criterion of 0.50. The internal consistency of the CIS was excellent, *α* = 0.90. Table 2 presents the factor loading and communalities of each item.

**Table 2:** Factor loadings and communalities obtained from exploratory factor analysis (N_1_ = 1076)

Item	Factor loading	Communalities (extraction)
1. Please indicate how much your current life is affected by the COVID-19 related problems.	.69	.47
2. Please indicate how much your current quality of life is damaged by the COVID-19 related problems.	.78	.61
3. How much are you worried about the COVID-19 related problems currently?	.56	.31
4. How often are you experiencing stress regarding the COVID-19 related problems currently?	.82	.67
5. How much are you experiencing fatigue regarding the COVID-19 related problems currently?	.75	.56
6. How much are you depressed by the COVID-19 related problems currently?	.68	.47
7. How often are you experiencing irritation regarding the COVID-19 related problems currently?	.76	.58
8. How often are you experiencing anger regarding the COVID-19 related problems currently?	.74	.55
9. How much do the COVID-19 related problems interfere with your interpersonal relationship?	.60	.36
10. How much do the COVID-19 related problems interfere with your studies, work, or household chores?	.61	.37

### Confirmatory factor analysis

A confirmatory factor analysis with maximum likelihood estimation was conducted on the second half of the whole sample (*N*_*2*_ = 1076) to confirm the single factor model of the CIS suggested in the EFA. Results revealed that the single factor solution had quite adequate fit, *χ*^*2*^(35) = 467.43, *p* < 0.001, comparative fit index (CFI) = 0.922, Tucker-Lewis fit index (TLI) = 0.900, goodness of fit index (GFI) = 0.916, normed fit index (NFI) = 0.917, non-normed fit index (NNFI) = 0.900, standardized root mean square residual (SRMR) = 0.044, and root mean square error of approximation (RMSEA) = 0.107 (90% CI: [0.099, 0.116]). Moreover, all standardized factor loadings were significant and ranged from 0.59 to 0.80 (see Table 3).

**Table 3:** Results from confirmatory factor analysis of the COVID-19 Impact Scale (N_2_ = 1076)

CFA for Items			CFA for Item Parcels		
Item	Standardized factor loading	Standard error	Item parcel	Standardized factor loading	Standard error
Item 1	.68***	.03	Item parcel 1	.81***	.02
Item 2	.76***	.03	Item parcel 2	.80***	.02
Item 3	.60***	.03	Item parcel 3	.86***	.02
Item 4	.80***	.02	Item parcel 4	.72***	.03
Item 5	.76***	.03			
Item 6	.75***	.03			
Item 7	.76***	.03			
Item 8	.75***	.03			
Item 9	.59***	.03			
Item 10	.64***	.03			

***p < .001

As noted above, the CFA results provided that fit indices indicated the suitability of the suggested single-factor model, except for the RMSEA. However, when a scale is lengthy, which means that there are more than five to eight items for each factor, the item-level analysis may make it difficult to confirm the results of the CFA [20]. In such cases, the use of item parcels, that is, using a sum of average of a set of items rather than individual items, is recommended [20, 38]. Therefore, we further conducted the CFA using the item parceling to examine the suitability of the single-factor model. Following these guidelines, we parceled items using a content-based parceling approach: general effect = items 1, 2 (item parcel 1); concerning and stressful reaction = items 3, 4 (item parcel 2); negative emotional reaction = items 5–8 (item parcel 3); interpersonal and functional effect = items 9, 10 (item parcel 4). We evaluated the model with a standard maximum-likelihood estimation. Fit indices indicated that the single factor model seemed to be a good fit to the data, *χ*^*2*^(2) = 16.77, *p* < 0.001, CFI = 0.993, TLI = 0.980, GFI = 0.992, NFI = 0.992, NNFI = 0.980, SRMR = 0.015, and RMSEA = 0.083 (90% CI: [0.049, 0.121]). RMSEA was slightly above 0.08 but within an acceptable range. Standardized factor loadings were all significant and ranged from 0.72 to 0.86 (see Table 3).

### Criterion validity and incremental validity

To examine the criterion-related validity of the CIS, we did correlation analysis with existing mental-health measures. As shown in Table 4, the scale was positively correlated with depression (*r* = 0.30, *p* < 0.001), anxiety (*r* = 0.28, *p* < 0.001), suicidal ideation (*r* = 0.08, *p* < 0.001), and fear of COVID-19 (*r* = 0.50, *p* < 0.001), and negatively correlated with subjective well-being (*r* = − 0.12, *p* < 0.001). The Fear of COVID-19 Scale did not show a significant correlation with suicidal ideation (*r* = − 0.019, ns) and subjective well-being (*r* = − 0.029, ns).

**Table 4:** Correlations between the COVID-19 Impact Scale and related scales (N = 2152)

	1	2	3	4	5	6
1. COVID-19 Impact Scale	–					
2. Fear of COVID-19 Scale	.50***	–				
3. Center for Epidemiologic Studies Depression	.30***	.12***	–			
4. Generalized Anxiety Disorder-7	.28***	.16***	.81***	–		
5. Mental Health Continuum-Short Form	− .12***	− .03	− .64***	− .47***	–	
6. Scale for Suicidal Ideation	.08***	− .02	.59***	.47***	− .49***	–
*M*	18.32	16.09	15.87	4.02	46.95	6.85
*SD*	7.42	4.72	12.14	4.33	13.50	5.21

***p < .001

To find out whether the CIS has an incremental explanatory power beyond that provided by the FCV-19S, we did hierarchical regression analysis. On CES-D and GAD-7, the FCV-19S was included in Step 1, and the CIS was added in Step 2. As shown in Table 5, the CIS produced a significant increase in variance that accounted for depression in Step 2 (*ΔR*^*2*^ = 0.08, *p* < 0.001), and the regression coefficient of the CIS was significant (β = *0.3*2*, p* < 0.001). However, the FCV-19S lost its significance (β = − 0.04, *p* = 0.13). Also, the CIS produced a significant increase in variance that accounted for anxiety in Step 2 (*ΔR*^*2*^ = 0.05, *p* < 0.001), and the regression coefficient of the CIS was significant (β = *0.2*6*, p* < 0.001). However, the FCV-19S also lost its significance (β = 0.03, *p* = 0.24).

**Table 5:** Hierarchical regression analysis on depression and anxiety (N = 2152)

		Depression (CES-D)			Anxiety (GAD-7)		
		β	*R*^*2*^	*ΔR*^2^	β	*R*^2^	*ΔR*^2^
1	Fear of COVID-19 Scale	.12***	.02	.02***	.16***	.03	.03***
2	Fear of COVID-19 Scale	− .04	.09	.08***	.03	.08	.05***
	COVID-19 Impact Scale	.32***			.26***		

***p < .001

## Discussion

The COVID-19 pandemic is prolonged. Content and severity of its effects around the world are diversifying. Given the current situation, we aimed to develop and validate a new scale, recognizing that the psychological effect of the COVID-19 pandemic should be identified considering various psychological difficulties. For these reasons, we constructed a measure for evaluating several negative emotions and quality of life deterioration due to COVID-19 pandemic-related problems to extend existing assessments. Exploratory factor analysis indicated a unidimensional structure with good psychometric properties. To examine the fitness of the suggested structure, we performed confirmatory factor analysis and found that the unidimensional model showed a good to excellent level of fitness. Correlation analysis with existing mental-health measures showed that the CIS had significant correlations with depression, anxiety, fear of COVID-19, suicidal ideation, and subjective well-being. Compared to the FCV-19S, which was developed early in the pandemic, the CIS was correlated with measures that the FCV-19S did not show correlations with. Moreover, hierarchical regression analysis on depression and anxiety revealed that the CIS had an incremental validity after controlling for the FCV-19S. These results suggest that the CIS is a valid and reliable scale for comprehensively assessing effects of the COVID-19 pandemic. It has better explanatory power than the existing scale.

Based on these findings, this study has some implications as follows. First, as far as we know, the CIS is the first scale developed to measure both negative emotional responses and difficulty in daily functioning relevant to the COVID-19 pandemic. In the current situation, where the COVID-19 pandemic is prolonged and its effects have become uneven, the lack of a valid tool to measure diversified effects of the COVID-19 pandemic can limit the ability to fully grasp the pandemic’s effects. In this regard, we believe that the CIS developed in this study will enable us to comprehensively identify and track the subjective effects of the COVID-19 pandemic.

Second, compared to the scale developed at the beginning of the pandemic, the CIS proved its inclusiveness by showing significant correlations with various mental-health measures tested in this study. In addition, although FCV-19S showed similar significance and direction of correlations, the explanatory power of the CIS was better. As reported in a previous study, the COVID-19 pandemic has resulted in various psychological reactions, although subjective well-being could not be sufficiently explained by identifying these psychological reactions by themselves, because there are additional factors, such as protective ones [48]. Therefore, the CIS, which showed wider and larger correlations with various negative emotions and quality of life deterioration, should enable convenient identification of comprehensive effects of the COVID-19 pandemic.

By integrating these implications, we can suggest specific applications as follows. Under the current the COVID-19 pandemic, psychological problems are considered to be a common stress response experienced by the majority of the general population. Not only the mental health professional field but also organizations of daily life such as companies and schools should pay attention to psychological problems of their members. Continuous and appropriate strategies are needed to identify and address members’ difficulties [6, 9]. In these situations, the CIS can be useful because it is short and easy to administer even for non-professionals while covering various emotional responses and functional difficulties.

The application of CIS seems not to be limited to non-clinical fields. In the COVID-19 pandemic, symptom-based screening is considered as one of key measures [39]. This can be applied not only to COVID-19 related respiratory symptoms but also to psychiatric primary care. In particular, there is an opinion that researchers and clinicians too often use clinical psychiatric measurement tools designed for pathologic disorders and validated using clinical populations to assess stress [29]. The CIS can be used to assess stress response specific to the COVID-19 pandemic without using psychiatric measurements.

Despite significances described above, limitations of this study and suggestions for subsequent studies are as follows. First, this study was limited in that the scale was validated only on college students. Given such sample limitations, sufficient representativeness could not be ensured, which could weaken the generalizability of our results. Therefore, the scale needs to be validated on various age groups and the general population. Second, our results reflected data from only one country. Since the lethality of COVID-19 differs around the world, as will its psychological effects, the scale needs to be validated in various countries. For example, mean scores of all items in this study were located in the range below moderate. Korea, where this study was conducted, has been able to control the pandemic without nationwide shutdown. Mean scores in the range below moderate may reflect this context specificity. To address this limitation, the scale needs to be validated in other countries.

## Conclusion

In this study, we aimed to develop and validate a scale to measure the stress response relevant to the COVID-19 (Corona virus disease 2019) pandemic comprehensively and concisely. The newly developed COVID-19 Impact Scale (CIS) was proven to be reliable and valid to measure various negative emotions and deterioration of quality of life caused by the COVID-19 pandemic. Moreover, the CIS showed better explanatory power than the existing scale developed early in the pandemic. From these results, we can assume that the CIS will be a useful simple scale to evaluate and monitor psychological difficulties due to the COVID-19 pandemic. The CIS seems to enable us to provide appropriate interventions during the immediate COVID-19 pandemic and to track problems that may persist beyond the end of the pandemic. Future research could validate the CIS in various subgroups and countries, and the comparison of CIS among various groups could help to understand the current psychological difficulties.

## Data Availability

The data that support the findings of this study are available at https://osf.io/24a7e/

## Appendix

*COVID-19 Impact Scale*

1. Please indicate how much your current life is affected by the COVID-19 related problems.
2. Please indicate how much your current quality of life is damaged by the COVID-19 related problems.
3. How much are you worried about the COVID-19 related problems currently?
4. How often are you experiencing stress regarding the COVID-19 related problems currently?
5. How much are you experiencing fatigue regarding the COVID-19 related problems currently?
6. How much are you depressed by the COVID-19 related problems currently?
7. How often are you experiencing irritation regarding the COVID-19 related problems currently?
8. How often are you experiencing anger regarding the COVID-19 related problems currently?
9. How much do the COVID-19 related problems interfere with your interpersonal relationship?
10. How much do the COVID-19 related problems interfere with your studies, work, or household chores?

The participants select the response that best describes their current state for each statement. A total score is calculated by summing up responses. It is based on 5-point Likert scale: None(0), mild/rarely(1), moderately/sometimes(2), severe/often(3), strongly severe/very often(4). The higher the score, the greater the impact of COVID-19 is, ranging from 0 to 40.

## Abbreviations

CES-D: Center for Epidemiologic Studies Depression
CFA: Confirmatory Factor Analysis
CFI: Comparative Fit Index
CIS: COVID-19 Impact Scale
COVID-19: Coronavirus Disease 2019
EFA: Exploratory Factor Analysis
FCV-19S: Fear of COVID-19 Scale
GAD-7: Generalized Anxiety Disorder 7-item
GFI: Goodness of Fit Index
JHU CSSE: Johns Hopkins University Center for Systems Science and Engineering
KMO: Kaiser–Meyer–Olkin
MHC-SF: Mental Health Continuum-Short Form
NFI: Normed Fit Index
NNFI: Non-Normed Fit Index
RMSEA: Root Mean Square Error of Approximation
SRMR: Standardized Root Mean square Residual
SSI: Scale for Suicide Ideation
TLI: Tucker-Lewis fit Index
WHO: World Health Organization
